# First description of immune complex vasculitis after COVID-19 vaccination with BNT162b2: a case report

**DOI:** 10.1186/s12879-021-06655-x

**Published:** 2021-09-16

**Authors:** Victoria Therese Mücke, Viola Knop, Marcus Maximilian Mücke, Falk Ochsendorf, Stefan Zeuzem

**Affiliations:** 1 Department of Internal Medicine 1, University Hospital Frankfurt, Theodor-Stern-Kai 7, 60590 Frankfurt am Main, Germany; 2 Department of Dermatology, Venereology and Allergology, University Hospital Frankfurt, Theodor-Stern-Kai 7, 60590 Frankfurt am Main, Germany

**Keywords:** Immune complex vasculitis, COVID-19 vaccination, Type three hypersensitivity, BNT162b2, Case report

## Abstract

**Background:**

Cases of immune complex vasculitis have been reported following COVID-19 infections; so far none in association with novel mRNA-based COVID-19 vaccination. This case report describes a cutaneous immune complex vasculitis after vaccination with BNT162b2.

**Case presentation:**

A 76-year old male with liver cirrhosis developed an immune complex vasculitis 12 days after the second injection of BNT162b2. On physical examination, the patient presented with pruritic purpuric macules on hands and feet, flexor and extensor parts of both legs and thighs and lower abdomen, and bloody diarrhoea. Laboratory testing showed elevated inflammatory markers. After short treatment with oral steroids all clinical manifestations and laboratory findings resolved.

**Conclusions:**

An increasing number of clinical manifestations have been attributed to COVID-19 infection and vaccination. This is the first written report of immune complex vasculitis after vaccination with BNT162b2. We present our case report and a discussion in the light of type three hypersensitivity reaction.

## Background

Patients infected with the novel corona virus disease of 2019 (COVID-19) prominently present with respiratory and thromboembolic complications. High infectivity and high mortality rates of this disease have lead—so far—to an incomparable fatal pandemic outbreak [1]. Shortly after the first description of COVID-19 in Wuhan, Hubei, China in December 2019, scientists started with the development of vaccines as a primary intervention strategy to control coronavirus transmission and infection [2]. Within a year, different vaccines have been developed to induce immunity in humans [3]. One of the earliest approved vaccines was the mRNA-based vaccine BNT162b2 [4]. By now, there have already been more than 1 billion COVID-19 vaccine doses administered worldwide [5]. Adverse events following COVID-19 vaccination mainly consist of typical vaccine related symptoms, such as pain at injection side, chills, fever, arthralgia, myalgia and headache [6]. We report of a patient who developed an immune complex vasculitis after vaccination with BNT162b2.

## Case presentation

**Fig. 1:**
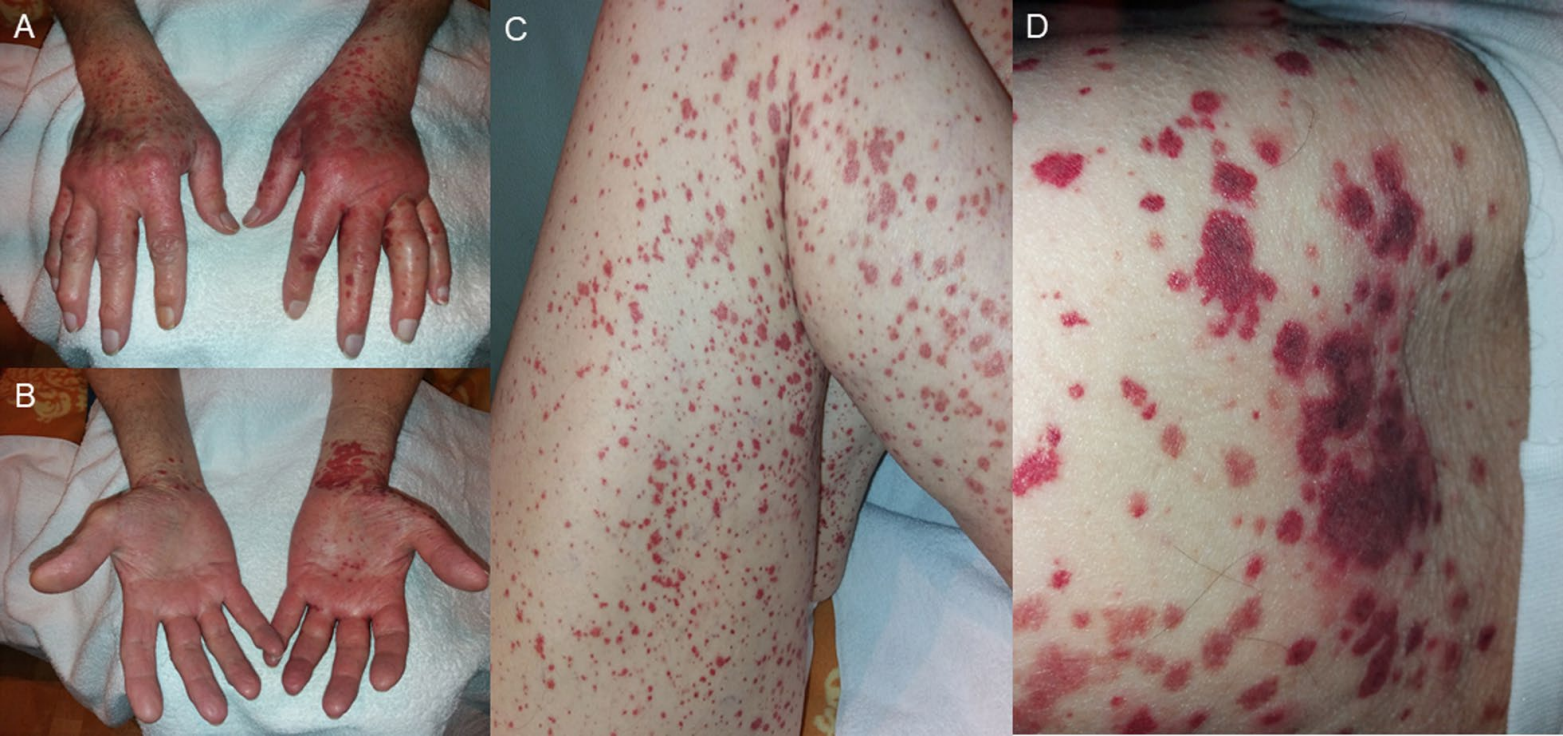
**A–D** Symmetric distal limb swelling and palpable purpuric confluent macules on extensor (**A**) and flexor (**B**) parts of both hands, legs (**C**) and thighs (**D**). Purpuric maculae ranging from 2 mm to few centimetres by confluence

A 76-year old Caucasian male with a history of compensated alcoholic liver cirrhosis, NYHA II heart failure, gastrectomy after gastroesophageal junction cancer and prostatectomy after prostate cancer and indwelling suprapubic catheter noticed lesions on hands and feet. Due to progressive pruritus and swelling, he shortly presented in our outpatient clinic of the Department of Internal Medicine, University Hospital Frankfurt. Physical examination revealed symmetric distal limb swelling, and a purpuric rash with palpable maculae on extensor and flexor parts of both hands, legs and thighs reaching up to the lower abdomen (Fig. 1). The smallest purpura lesions were a few millimetres in size and confluent up to centimetres in size. The lesions persisted on diascopy. Upon presentation, the patient denied symptoms of fever, dyspnoea, arthralgia or anuria, but he reported of melaena and diarrhoea the day before. The patient had no history of allergic predispositions or systemic autoimmune diseases. He denied to have started any new medication recently or any changing in alimentary habits. Yet, the patient reported that 12 days ago he received his second mRNA-based vaccination for COVID-19 with BNT162b2. Interestingly, the described skin findings were not seen on his last regular visit 2 days before the second vaccination. After his first vaccination with BNT162b2 6 weeks earlier, he had experienced significant vaccine related myalgia, fever (39.5 °C), hoarseness and fatigue, yet no skin related adverse events had occurred. Before that, he had never experienced any adverse events on previous vaccinations including influenza, herpes zoster or pneumococcal vaccinations.

Laboratory findings revealed elevated blood sedimentations rate, interleukin-6 levels and C-reactive protein levels. In addition, signs of discrete haemolysis and elevated ammonium levels could be detected. Known moderate micro-erythruria and leukocyturia did not worsen, but stool tests on occult blood was positive and stool calprotectin levels were moderately elevated. Virological tests on human herpes viruses and further human pathogenic viruses showed no new reactivation or infection (Table 1).

**Table 1:** Patient’s laboratory parameters

Parameter	2 days before 2nd vaccination	12 days after 2nd vaccination	5 days later on prednisolone	Normal range
Blood results				
CRP (mg/dl)	2.09	**8.69**		< 0.50
Creatinine (mg/dl)	0.82	0.97	1.43	0.70–1.20
Albumin (g/dl)	3.5	3.5	3.1	3.5–5.2
Total bilirubin (mg/dl)	1.0	1.4	0.7	< 1.4
Direct bilirubin /mg/dl)		0.8	0.4	< 0.3
ASAT (U/l)	47	36	51	< 40
ALAT (U/l)	38	25	37	< 50
GGT (U/l)	31	44	69	< 50
LDH (U/l)	205	211	223	< 248
CK (U/l)	85	108		< 190
Lactate (mg/dl)		16.3		4.5–20.0
ESR (mm/h)		**42**	**19**	< 20
PCT (ng/ml)		0.29		< 0.50
IL-6 (pg/ml)		**104.0**	**26.6**	< 7.0
Leucocytes (/nl)	4.57	5.87	6.01	3.92–9.81
Haemoglobin (g/dl)	11.6	11.3	10.1	13.5–17.5
Platelets (/nl)	141	114	133	146–328
INR	1.19	1.23	1.26	0.85–1.27
IgG (mg/dl)	1567	1549	1364	700–1600
C3c (mg/dl)			87	90–180
C4 (mg/dl)			16.1	10.0–40.0
ANA		1:80		< 1:80
c-ANCA (IFT)		Negative		Negative
p-ANCA (IFT)		Negative		Negative
c-ANCA (PR3) EILSA		Negative		Negative
p-ANCA (MPO) ELISA		Negative		Negative
Urine results				
Leukocytes (/µl)		350	447	< 10
Erythrocytes (/µl)		11	30	< 5
Stool results				
Calprotectin (µg/g)		**115**		< 50
EDN (ng/ml)		802		< 1700
iFOBT		**Positive**		Negative
Stool pathogens		Negative		
Virology				
CMV-DNA		Negative		
EBV-DNA		Negative		
HSV-1 IgG		positive 17.1 index		
HSV-2 IgG		Negative		
HHV-6 IgG		Negative		
HHV-6 IgM		Negative		
VZV IgG		Positive 2727 mE/ml		
VZV IgM		Negative		
Parvovirus B19 IgG		Positive 10 index		
Parvovirus B19 IgM		Negative		
Microbiology				
Treponema pallidum screening		Negative		
Mycoplasma pneumoniae IgG		Negative		
Mycoplasma pneumoniae IgM		Negative		

Newly elevated laboratory results and dynamics are presented in bold

*CRP* c-reactive-protein, *ASAT* aspartate transferase, *ALAT* alanine transferase, *GGT* gamma glutamyl transferase, *LDH* lactate dehydrogenase, *CK* creatinine kinase, *ESR* erythrocyte sedimentation rate, *PCT* procalcitonin, *IL* interleukin, *INR* international normalized ratio, *IgG* immunoglobulin G, *C3C* complement component 3, *C4* complement component 4, *ANA* antinuclear antibody, *ANCA* antinuclear cytoplasm antibody, *EDN* eosinophil-derived antitoxin, *iFOBT* immunochemical faecal occult blood test, *CMV* cytomegalovirus, *DNA* desoxyribonucleic acid, *Ig* immunoglobulin, *EBV* Ebstein-Barr-virus, *HSV* herpes simplex virus, *HHV* human herpes virus, *VZV* varicella zoster virus

**Fig. 2:**
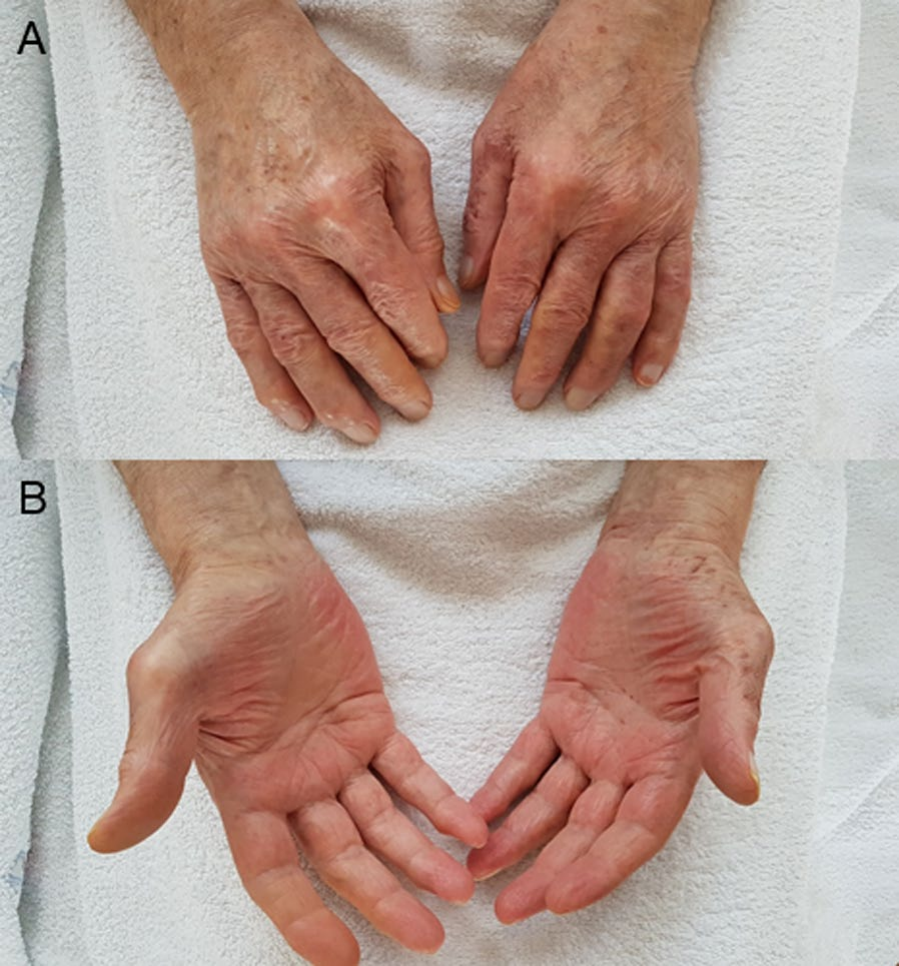
**A**, **B** Resolved skin lesions of extensor (**A**) and flexor (**B**) parts of both hands on the fifth day of steroid therapy

In consultation with our senior physician specialist from the Department of Dermatology, Venereology and Allergology, the correlation of the clinical presentation, laboratory findings and recent COVID-19 vaccine, we established the diagnosis of cutaneous and gastrointestinal immune complex vasculitis probably triggered by BNT162b2 injection. The patient was started on 40 mg oral prednisolone once daily. Shortly after, the patient reported of symptom relief. On the 5th day of therapy, he already presented with rapid resolving skin lesions (Fig. 2). At follow-up visit 23 days after BNT162b injection, we could observe small remaining scabs on both lower legs and the patient described remaining hyperalgesia while touching these lesions. Oedema, pruritus and purpuric lesions fully resolved and inflammation markers decreased. Stool formation and colour returned to normal. Prednisolone was quickly tapered and stopped.

## Discussion and conclusions

Despite of rapid pulmonary failure, vascular complications due to coagulopathy, endotheliopathy and vasculitis significantly contribute to the high mortality rates of patients infected with severe acute respiratory syndrome coronavirus type 2 (SARS-CoV2) [7]. Recent observations also showed that infections with SARS-CoV-2 may go along with mild to fulminant dermal diseases [8]. Until now, data on pathogenesis and clinical manifestations of COVID-19 support the hypothesis of three distinct phases of the disease: viral phase, immunological phase and haemo-vascular phase [9, 10]. In October 2020 Manzo discussed COVID-19 as a trigger for an immune complex hypersensitivity reaction [11]. He highlighted the significant antigen excess in the second phase of COVID-19 disease that may lead to the formation of soluble antigen-antibody immune complexes, which cannot be removed by phagocytes anymore. These may settle and persist in tissue places, e.g., skin where they can induce persistent inflammation. In general, multiple **i**nfectious diseases are known to be facultative triggers of immune complex diseases, e.g., beta-hemolytic streptococcal bacteria that precede purpura Schoenlein-Henloch disease in pediatric patients [12], an example of type three hypersensitivity. Interestingly, a recent letter to the editor of Roncati et al. discussed COVID-19 preceding a type three hypersensitivity vasculitis by inducing a severe inflammatory state by an interleukin-6 mediated cytokine release syndrome [13].

As vaccines are supposed to induce antigen-antibody reactions, novel COVID-19 vaccines mediated by the SARS-CoV2-spike-protein may trigger similar pathogenic effects. So far, cutaneous immune complex vasculitis have been seen in older patients after receiving seasonal influenza vaccinations [14]. Until now, data on the range of side effects of the novel COVID-19 vaccines are rare, but especially severe thrombotic [15] and anaphylactic reactions [16] drew scientific and general society’s’ attention. Recently, Bomback et al. published a summary of reported glomerular diseases after COVID-19 vaccination, including ten cases of IgA nephropathies [17]. MRNA-based vaccines have never been licensed for application in humans before this pandemic. Therefore, effects and side effects of BNT162b2 and its related agents are of great scientific interest. In current literature safety and efficacy of BNT162b2 was similar to other viral vaccines [18].

Our case report is the first to describe a cutaneous and gastrointestinal immune complex vasculitis after a mRNA-based vaccination with BNT162b2, in a cirrhotic patient without any history of autoimmune or vasculitis predisposition. Immune complex related extrahepatic conditions can be seen in patients with chronic liver disease. However, the majority of these cases are described in patients with liver disease due to hepatitis C or B infection [19, 20] or florid bacterial infection [21]. One can assume that a chronic liver disease may have precipitating effects of the deposition of immune complexes with defective liver metabolism of IgA circulating immune complexes, but we interpret the mRNA-vaccination as the main trigger of this phenomenon in our patient.

In general, most cases of cutaneous immune complex vasculitis are self-limited [22]. As our patient presented with pronounced skin involvement, additional gastrointestinal inflammation and bleeding signs we decided to treat him with oral anti-inflammatory steroids. Thereby, we achieved fast relief and restoration of our patient’s health. In patients with severe organ failure, strong immunosuppressive therapy and plasmapheresis may be necessary [23].

We acknowledge that the link between BNT162b2-vaccine and cutaneous and gastrointestinal immune-complex vasculitis cannot be confirmed by a single case. However, our theory is supported by: (1) the fact that the patient did not have any history of vasculitis before—and no dermal lesion was seen 2 days prior the second vaccination, (2) the suitable time span of 12 days between exposition and appearance of type three hypersensitivity reaction without any hints of an alternative cause—no changes in medication, alimentary habits or sign of another infection (3) interleukin-6 mediated inflammatory response and (4) data on COVID-19 infections that trigger immune complex vasculitis.

Worldwide, more than one billion COVID-19 vaccines doses have been injected to control the SARS-CoV-2 pandemic [5]. Among these, novel mRNA-based vaccines are administered on large scale for the first time in history. So far, serious adverse events reported with mRNA-vaccines, especially BNT162b2 are rare and efficacy rates are high [18]. However, clinical trials will not be able to detect rare clinical side effects. We are the first to describe the event of an immune complex vasculitis in a patient with liver cirrhosis without known predispositions for type 3 hypersensitivity. The purpose of this case report is to raise awareness of possible side effects in healthcare professionals in the so far world’s fastest and biggest vaccination program. We aim to highlight the importance of further scientific and clinical vigilance and surveillance.

## Data Availability

All data generated or analyzed during this study are included in this published article. More information are available from the corresponding author on reasonable request. Identifying/confidential patient data will not be shared.

## Abbreviations

COVID-19: Corona virus disease of 2019
SARS-CoV2: Severe acute respiratory syndrome coronavirus type 2
